# Reactive Molecular Dynamics Study of Pollutant Formation Mechanism in Hydrogen/Ammonia/Methanol Ternary Carbon-Neutral Fuel Blend Combustion

**DOI:** 10.3390/molecules28248140

**Published:** 2023-12-17

**Authors:** Jingyun Sun, Qianqian Liu, Yang Wang, Mingyan Gu, Xiangyong Huang

**Affiliations:** 1 School of Energy and Environment, Anhui University of Technology, Ma’anshan 243002, China; jingyunsun133@163.com (J.S.); liuqianqian208dw@163.com (Q.L.); gumy@ahut.edu.cn (M.G.); huangxy@ahut.edu.cn (X.H.); 2 School of Materials Science and Engineering, Anhui University of Technology, Ma’anshan 243032, China

**Keywords:** carbon-neutral fuel, ternary blend combustion, NO_X_, ReaxFF MD

## Abstract

Hydrogen, ammonia, and methanol are typical carbon-neutral fuels. Combustion characteristics and pollutant formation problems can be significantly improved by their blending. In this paper, reactive molecular dynamics were used to investigate the pollutant formation characteristics of hydrogen/ammonia/methanol blended fuel combustion and to analyze the mechanisms of CO, CO_2_, and NO_X_ formation at different temperatures and blending ratios. It was found that heating can significantly increase blending and combustion efficiency, leading to more active oxidizing groups and thus inhibiting N_2_ production. Blended combustion pollutant formation was affected by coupling effects. NH_3_ depressed the rate of CO production when CH_4_O was greater than 30%, but the amount of CO and CO_2_ was mainly determined by CH_4_O. This is because CH_4_O provides more OH, H, and carbon atoms for CO and CO_2_ to collide efficiently. CH_4_O facilitates the combustion of NH_3_ by simplifying the reaction pathway, making it easier to form NO_X_.

## 1. Introduction

Currently, the global transportation industry relies mainly on fossil energy sources [1], but the combustion of these traditional fossil energy sources causes a lot of pollution. To fundamentally solve this problem, finding clean energy sources that can replace traditional energy sources has become one of the most important research topics [2,3].

H_2_ and NH_3_ are both ideal clean and renewable fuels that have received a lot of attention from scholars at home and abroad. H_2_ can be produced renewably from green energy by electrolyzing water. In addition, it is characterized by good combustibility, low ignition energy, and fast combustion rates [4,5]. However, difficulties in storage and transportation, its excessive combustion rate, and its high combustion temperature producing NO_X_ pollution have limited the practical promotion of pure H_2_ fuel use [6]. NH_3_, as a good zero-carbon H_2_ storage carrier, can be obtained from fossil fuels, biomass, or other renewable sources. This is why NH_3_ has received a great deal of attention from the combustion community in recent years and is considered a sustainable fuel that can be remotely transported and applied [7]. NH_3_ is currently used as a fuel in a wide range of applications, such as vehicle engines [8], marine engines [9], and combustion engines for power generators [10]. The low viscosity of NH_3_ helps in fuel atomization and droplet formation during fuel injection [11]. In addition, NH_3_ has a high octane rating, which makes it suitable for engines with high compression ratios and reduced detonation [12]. However, NH_3_ has the disadvantages of a low combustion rate [13], high autoignition temperature [14], and narrow flammability limits, often leading to incomplete combustion. This contributes to poor engine performance, making it difficult to use as a single fuel for direct combustion [15,16]. The use of H_2_ as a combustion aid and NH_3_ blending has been found to be one of the ways to improve the efficiency of NH_3_ combustion [17]. This not only leads to improved in-cylinder combustion [18] but also reduces the requirement for engine modifications (material compatibility), thus ensuring a cost-effective transition to H_2_ [19]. Wang et al. [20] found that engine exhaust heat can crack some of the NH_3_ into H_2_ and nitrogen to provide energy, making this method much more maneuverable. However, a study by Alam et al. [21] pointed out that although H_2_-NH_3_ blending reduces carbon emissions, including CO, etc., in diesel internal combustion engines, incomplete fuel combustion and higher NO_X_ were observed.

Blending oxygenated fuels as combustion aids is also an effective way to improve combustion performance and pollutant emissions in diesel engines [22]. In a study of LPG-diesel- and CNG-diesel-fueled diesel engines using the high-cetane fuel diethyl ether, improved combustion was observed [23]. Wang et al. [24] performed numerical simulations of ethanol and diesel blends on their combustion and emission characteristics. It was found that ethanol/diesel blends significantly reduced CO_2_ and soot emissions compared to diesel. Soot and CO_2_ emissions were reduced by 63.25% and 17.24% respectively at 100% load, but Nox was increased by 1.39%. Feng et al. [25] analyzed a methanol/diesel/n-butanol replacement blend. The results show that the thermal efficiency and the blending efficiency of diesel and alcohol fuel increase with an increase in the alcohol fuel blending ratio (0–15%), and irreversible loss also increases. Increasing the load on a diesel engine can improve its thermal efficiency. Wang et al. [26] investigated DGE as an oxygenated fuel and combustion enhancer to improve the combustion emissions of NH_3_ and H_2_ blends. It was found that when 60–70% of diesel fuel was replaced with DGE, H_2_, and NH_3_, CO_2_ was reduced by 50% and synergistic effects were found between DGE and H_2_ and NH_3_, reducing PM, NO_X_, HC, and CO emissions.

CH_4_O, as the saturated monohydric alcohol with the simplest structure, is inexpensive and simple to synthesize. It is a high-quality representative for the study of combustion-enhancing effects on oxygenated fuels. Li et al. [27] found that blending a small amount of CH_4_O into NH_3_ combustion made the blend more reactive, due to the enrichment of the O/H radical pool by the addition of CH_4_O. Species in this sequence can also react directly with NH_3_ combustion-associated species, thereby consuming NH_3_ and promoting spontaneous combustion. Xu et al. [28] simulated the combustion characteristics of NH_3_/CH_4_O blends and found that CH_4_O makes a significant contribution to the laminar combustion rate of NH_3_, and NO_X_ emission analysis showed that the blending of 60% CH_4_O leads to the highest NO_X_ emissions. Lu et al. [29] investigated the effect of CH_4_O doping on NH_3_ combustion and emissions by modeling the chemical reaction mechanisms of an NH_3_/CH_4_O blend. The results showed that CH_4_O doping significantly increased the chemical reaction activity of NH_3_ and significantly reduced the ignition delay time.

Because of the complexity of the engine in cylinder combustion and its pollutant formation characteristics, it is not favorable to explore the chemical reaction kinetics and blended fuel combustion pollutant laws under different operating parameters in isolation [30,31]. In this paper, CH_4_O is used as a representative of oxygenated fuels. Reactive molecular dynamics are used to investigate the effect of CH_4_O on the combustion pollutant formation characteristics of H_2_ and NH_3_ combustion-reforming gases in diesel engines. This study analyzes the pollutant formation mechanisms of CO, CO_2_, and NO_X_ formations at different temperatures and fuel ratios at the molecular level. This study is of great theoretical and practical significance to enhance the application of carbon-neutral fuels in engines and other practical combustion equipment.

## 2. Results and Discussion

### 2.1. Temperature Effects on Ternary Blended Combustion Components and Pollutant Formation

#### 2.1.1. Temperature Effects on Ternary Blended Combustion Components and Free Radicals

**Figure 1:**
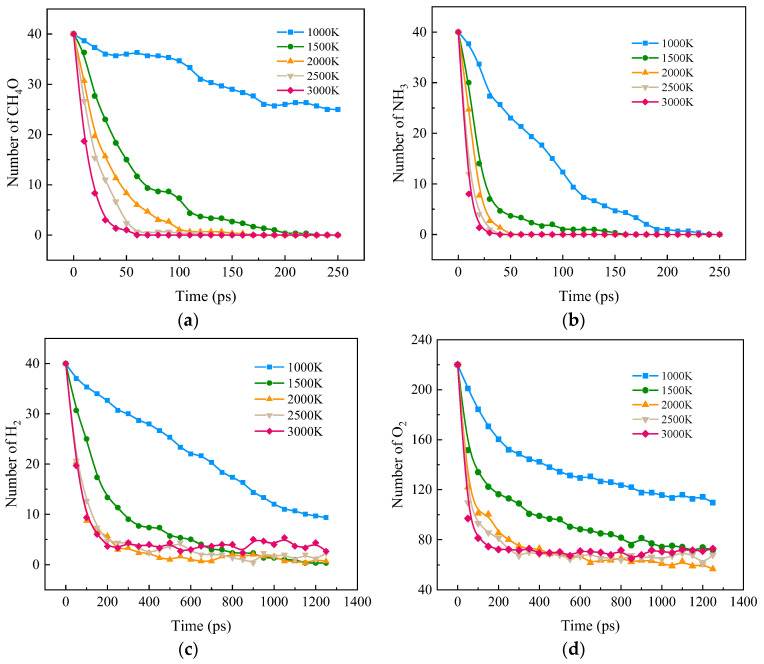
Changes in reactants during the combustion of carbon-neutral fuels at different temperatures. (**a**) CH_4_O; (**b**) NH_3_; (**c**) H_2_; (**d**) O_2_.

Figure 1 shows the effects of different temperatures on the four reactant components CH_4_O, NH_3_, H_2_, and O_2_ in the ternary blended combustion process. From the figure, it can be seen that heating significantly accelerated the decomposition rate of CH_4_O, NH_3_, H_2_, and O_2_. The insignificant rises for H_2_ and O_2_ at high temperatures may be caused by the decomposition of H_2_O due to the intensification of molecular collisions at high temperatures.

**Figure 2:**
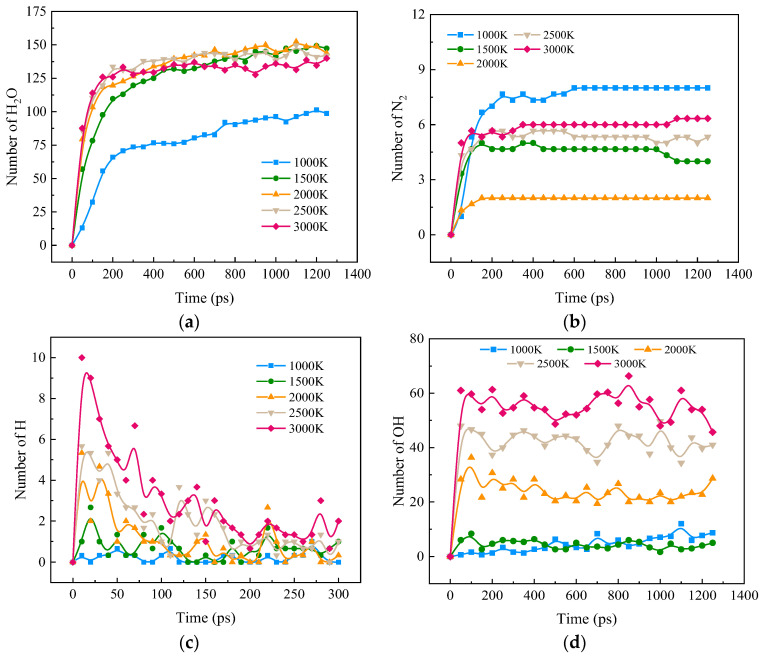
Changes in components and radicals during combustion at different temperatures. (**a**) H_2_O; (**b**) N_2_; (**c**) H; (**d**) OH.

Figure 2a,b shows the effects of different temperatures on the formation of H_2_O and N_2_ in the combustion process. From Figure 2a, it can be seen that the growth rate of H_2_O slows down significantly after a rapid increase to a certain level. Heating accelerates the rate of H_2_O formation during combustion. However, the effect of heating is not obvious when the temperature is further increased above 2000 K. Above 2500 K, H_2_O shows an insignificant decreasing trend, which may be due to the decomposition of H_2_O at high temperatures. This conclusion is consistent with the above conclusion that high temperatures lead to a slowly increasing trend for H_2_ and O_2_ at the late stage of the reaction. In Figure 2b, it is visible that the variation rule for N_2_ at different temperatures is not strictly temperature-dependent. The maximum amount of N_2_ generated by the reactants is at 1000 K. Above 2500 K and 1500 K, the amount of N_2_ generated increases with an increase in temperature. However, in the case of 2000 K, the amount of N_2_ is significantly lower than for other temperatures. This is because N from NH_3_ generates more NO_X_ at 2000 K.

Figure 2c,d shows the effects of different temperatures on the formation of H and OH during blended combustion. It can be seen that the formation of H and OH is slow at low temperatures and the quantity is depleted as the reaction continues. High temperatures increase the amounts of H and OH. The difference is that H peaks rapidly and then decreases as the reaction proceeds, while OH peaks and then stabilizes as the reaction proceeds. The peak H at 3000 K is five times higher than that at 1500 K. Heating significantly increases the H and OH concentrations in the combustion reaction.

#### 2.1.2. Temperature Effects on CO and CO_2_ Formation in Blended Combustion

**Figure 3:**
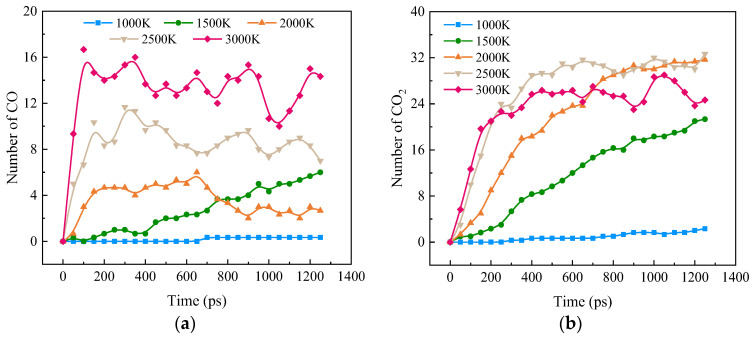
CO and CO_2_ formation with time for blended combustion at different temperatures (**a**) CO; (**b**) CO_2_.

Figure 3a,b shows CO and CO_2_ formation during the blended combustion process at different temperatures. Heating increases the rate of CO production, where CO is formed rapidly and then decreases slowly at temperatures of 2000 K and higher. At 1500 K and 1000 K, CO has still not peaked at the end of the reaction and is in a state of continuous growth. As the temperature increases the CO_2_ production rate increases, and the peak state remains almost stable. However, some of the CO is further oxidized to CO_2_ at high temperatures. Heating significantly accelerates the production of CO and CO_2_. The decrease in CO_2_ at 3000 K is because the high temperature promotes the reduction of more CO_2_ to CO.

#### 2.1.3. Temperature Effects on NO_X_ Formation from the Blended Combustion of Ternary Carbon-Neutral Fuels

**Figure 4:**
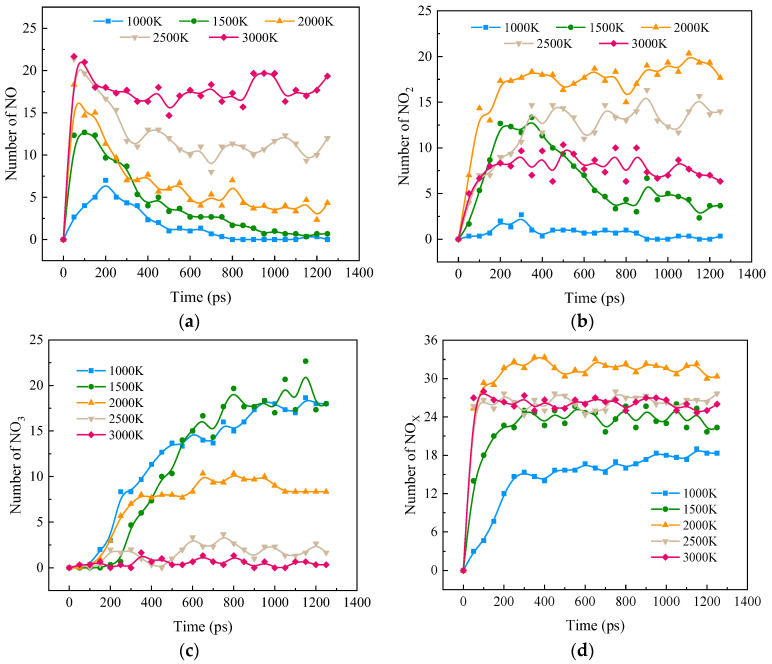
Distribution of NO_X_ during the combustion of ternary carbon-neutral fuel blends. (**a**) NO; (**b**) NO_2_; (**c**) NO_3_; (**d**) NO_X_.

Figure 4 shows the effects of temperature on the formation of NO_X_ (NO, NO_2_, and NO_3_) in the combustion of ternary carbon-neutral fuel blends. From Figure 4a, it can be seen that, as the reaction proceeds, NO is first generated rapidly. NO at low temperatures is gradually depleted after reaching the peak value, but the amount of NO at high temperatures is relatively stable. As the temperature increases, the NO peak is gradually shifted forward, and the peak value increases.

Figure 4b shows the change in NO_2_ with combustion, and its change rule in the range of 2000 K to 3000 K is opposite to that of NO, in which the largest amount of NO_2_ exists at 2000 K, followed by 2500 K, with the least at 3000 K, which may be caused by part of NO_2_ being reduced at a high temperature. NO_2_ at 1500 K shows the same trend of increasing and then decreasing as NO in this condition. The time of peak NO coincides with the time of a rapid increase in NO_2_, and the time of a large amount of NO consumption coincides with the time of peak NO_2_, so the consumed NO_2_ is further oxidized to NO_3_.

Figure 4c represents the variation in NO_3_ as the combustion reaction proceeds, with almost no change for the two high-temperature conditions. Its rise at 2000 K is followed by a steady rise and a rapid and sustained rise in the low-temperature condition.

Figure 4d represents the rapid formation and gradual stabilization of NO_X_ as the blended combustion reaction proceeds. The effect of heating on the NO_X_ peak is nonlinear. The NO_X_ peak growth rate slows down with increasing temperature and reaches a peak at 2000 K. The NO_X_ peak growth rate is also shown in Figure 4d, which shows that NO_X_ formation is rapid and stabilizes under ternary combustion.

Heating accelerates the formation of NO_X_, but high temperature inhibits the formation of NO_X_ when the temperature is higher than 2000 K. At low temperatures, NO_X_ exists mainly in the form of NO_3_. At high temperatures, the main form of NO_X_ is NO. At 2000 K, NO_2_ is the main form of NO_X_. This is probably because high temperature accelerates the reduction of NO_X_.

### 2.2. Influence of Blending Ratio on Combustion Composition and Pollutant Formation

#### 2.2.1. Influence of Blending Ratio on Combustion Components and Free Radicals

**Figure 5:**
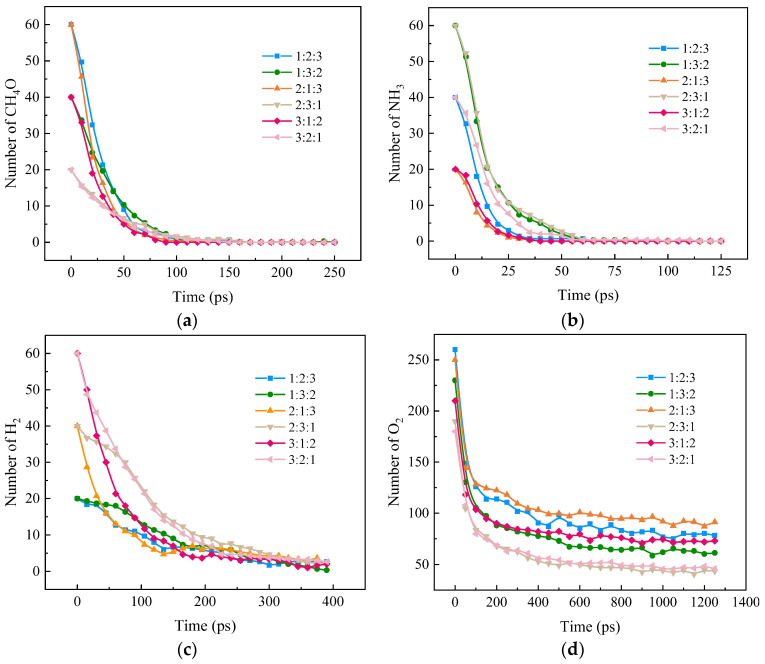
Changes in reactants in the combustion process of ternary carbon-neutral fuel blends with different blending ratios. (**a**) CH_4_O; (**b**) NH_3_; (**c**) H_2_; (**d**) O_2_.

A comparison and analysis of different blending ratios were carried out to obtain the formation patterns of reactants and NO_X_ in ternary blended combustion under different blending ratios. Figure 5 shows the changes in reactants with time under different blending ratios. When the proportion of CH_4_O is more than 30%, the lower the proportion of NH_3_, the faster and more complete the reaction. When the proportion of CH_4_O is less than 30%, the reaction rate of CH_4_O is faster in the case of H_2_/NH_3_ being more than 1, which is because H_2_ promotes the decomposition of CH_4_O. Therefore, NH_3_ inhibits CH_4_O combustion and H_2_ promotes CH_4_O combustion in blended fuel combustion. When the proportion of CH_4_O is more than 30%, NH_3_ plays a major role. When the proportion of CH_4_O is less than 30%, H_2_ plays a major role.

As shown in Figure 5b, the higher the CH_4_O percentage, the higher the NH_3_ reaction rate and the more complete the reaction. When the amount of NH_3_ is determined, the working NH_3_ reaction rate is faster for CH_4_O/H_2_ greater than 1. This is because CH_4_O can promote the combustion of NH_3_. As shown in Figure 5c, the lower the NH_3_ percentage the higher the H_2_ reaction rate and the more complete the reaction. This is because NH_3_ inhibits H_2_ combustion. When the amount of H_2_ is determined, the H_2_ reaction rate is faster for the working condition of CH_4_O/NH_3_ greater than 1, which also indicates that NH_3_ inhibits H_2_ combustion during the combustion of blended fuels.

**Figure 6:**
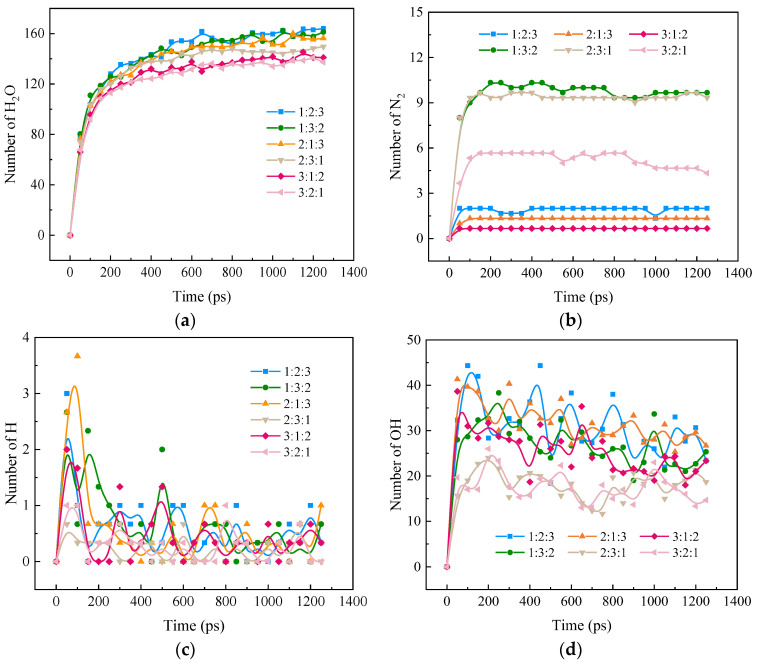
Variation in combustion components and free radicals in ternary carbon-neutral fuel blends with time at different blending ratios. (**a**) H_2_O; (**b**) N_2_; (**c**) H; (**d**) OH.

From Figure 6a, it can be seen that the growth rate of H_2_O slows down significantly after a rapid rise to a certain level. With the increase in the proportion of H_2_, the rate of H_2_O generation is accelerated, and it can be seen that H_2_ accelerates the rate of H_2_O formation in the combustion process. Through Figure 6b, it can be found that with the reaction, N_2_ rises rapidly to a certain degree and then stabilizes, and N_2_ rises with an increase in NH_3_ content under different doping ratios. Because H_2_ and NH_3_ have a competitive relationship in the combustion process, and N_2_ is a product of NH_3_ combustion, when NH_3_ is the same, the smaller the proportion of H_2_ the faster N_2_ rises and the larger the peak. However, in the case of a H_2_/NH_3_/CH_4_O ratio of 1:2:3, the amount of N_2_ is significantly lower than a ratio of 3:2:1, which may be due to the large amount of CH_4_O affecting the conversion of NH_3_ to N_2_ during combustion, which will be further verified at the molecular level in Section 3.4.

Figure 6c,d, on the other hand, shows the effect of different blending ratios on the formation of H and OH during the ternary hybrid combustion. It can be seen that H shows an increasing and then decreasing trend as the reaction proceeds. The conclusion is that the peak H value increases with increasing CH_4_O percentage. When the proportion of CH_4_O in the blending fuel remains constant, H_2_ has a certain promotion effect on H peak generation, and NH_3_ has a certain inhibition effect. When the proportion of CH_4_O is more than half, the inhibitory effect is greater than the promotion effect, and OH grows to the peak and then decreases slowly as the reaction, which is almost stable, proceeds. The peak rises with the increase in CH_4_O percentage. When the proportion of CH_4_O in the blending fuel remains constant, unlike H, NH_3_ promotes the generation of OH while H_2_ inhibits it, and the inhibitory effect is greater than the promotional effect when the proportion of H_2_ is more than half. Therefore, the concentration of radicals H and OH in the ternary blended combustion reaction is mainly affected by CH_4_O.

#### 2.2.2. Influence of the Blending Ratio on CO and CO_2_ Formation in Blended Combustion

**Figure 7:**
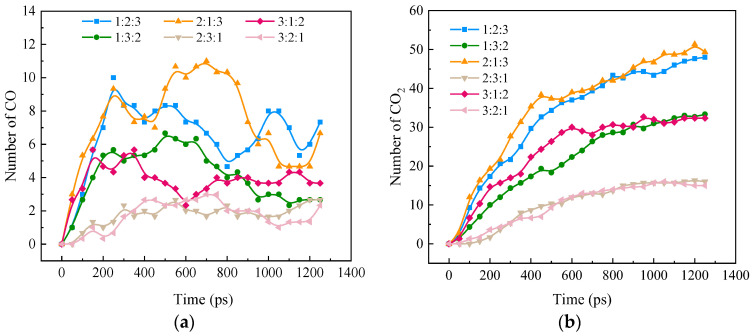
CO and CO_2_ formation over time for blended combustion at different blending ratios (**a**) CO; (**b**) CO_2_.

Figure 7a,b shows CO and CO_2_ formation during the ternary fuel blending process at different blending ratios, respectively. As the CH_4_O combustion reaction proceeds, the rate of CO formation is mainly affected by NH_3_, which slows down the rate of CO formation at a CH_4_O share of more than 30%. However, the amount of CO production is mainly influenced by CH_4_O, which increased with the increase in CH_4_O percentage. When the proportion of CH_4_O in the blending fuel remains constant, the larger the proportion of H_2_, the larger the peak of CO, which may be due to the combustion process of H_2_ to promote the production of CO. CO_2_, in the progress of the reaction, shows a continuous increase in the trend of the rate, and the amount of CO_2_ production is mainly affected by the proportion of CH_4_O. When the proportion of CH_4_O in the blending fuel remains constant, the higher the proportion of H_2_, the faster the reaction, and the greater the amount of formation, indicating that H_2_ plays a role in promoting the formation of CO_2_, while NH_3_, and CH_4_O have a competitive relationship.

In summary, it is shown that the production of CO and CO_2_ during the combustion of a blend of ternary carbon-neutral fuels is not simply influenced by CH_4_O alone but is a result of the coupling of three fuels, H_2_, NH_3_, and CH_4_O, which will be examined on a molecular level in a detailed pathway analysis in Section 3.4.

#### 2.2.3. Influence of Blending Ratio on NO_X_ Formation in Blended Combustion

**Figure 8:**
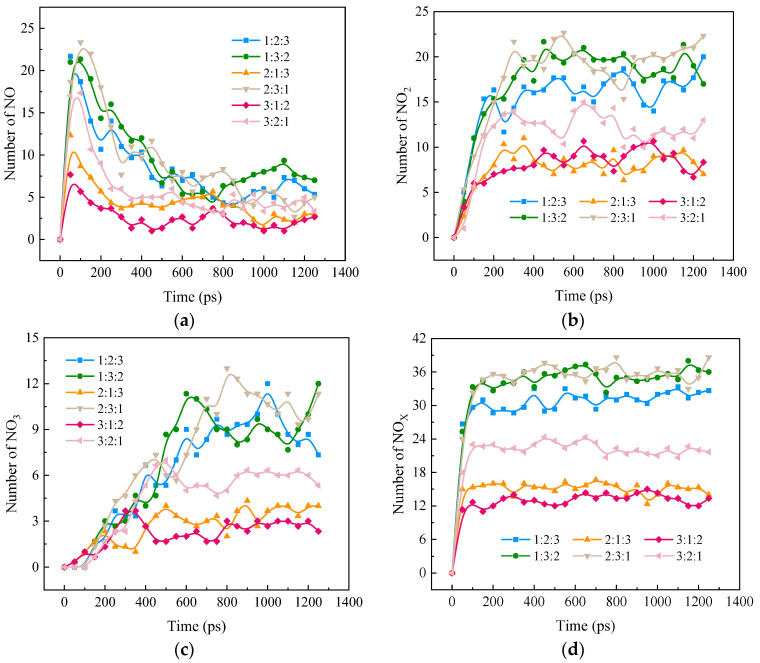
Distribution of NO_X_ during the combustion of ternary carbon-neutral fuel blends at different blending ratios. (**a**) NO; (**b**) NO_2_; (**c**) NO_3_; (**d**) NO_X_.

Figure 8 shows the influence of the NH_3_ blending ratio on the formation of NO_X_ (NO, NO_2_, and NO_3_) during the blended combustion of ternary carbon-neutral fuels. Figure 8a shows that, as the reaction proceeds, NO is first generated rapidly and then gradually depleted. The NO formation rates and the peak values are in the ratios 2:3:1, 1:3:2, 1:2:3, 3:2:1, 2:1:3, and 3:1:2 from large to small, respectively. The time of peak appearance is positively correlated with the size of the peaks. The rate of NO formation and the magnitude of the peak are mainly influenced by NH_3_ in the fuel blends. When NH_3_ is quantized, CH_4_O promotes NH_3_ combustion. Figure 8b represents the change in NO_2_ with the combustion reaction process, and the NO_2_ reaction fluctuates up and down around the peak value after a certain stage. The trend of the magnitude of the stabilization value with the blending ratio is similar to that of NO, and the rate of NO_2_ formation and the magnitude of the peak are mainly affected by the NH_3_ in the blended fuels. Comparing 1:2:3 and 3:2:1, it can be seen that CH_4_O promotes the combustion of NH_3_ but increases the formation of NO_2_. Figure 8c represents the variation in NO_3_ as the combustion reaction proceeds, showing a continuous growth trend as the reaction proceeds, but the growth rate is slow and then fast. The variation in the blending ratio is also similar to that of NO.

Figure 8d shows the rapid formation and gradual stabilization of NO_X_ as the combustion reaction proceeds. As the NH_3_ percentage increases, the NO_X_ peak increases. When NH_3_ is quantized, the higher the CH_4_O content, the higher the NO_X_ peak. Based on the NH_3_ percentage, it was hypothesized that there should be little difference between the ratio of 1:2:3 and the ratio of 3:2:1 NO_X_ quantities, but the result was unexpected. The NO_X_ value of 1:2:3 was 25% higher than that of 3:2:1, which may be due to the fact that CH_4_O increased the conversion rate of NH_3_ to NO_X_. In order to gain insight into the effects of doping ratio on NO_X_ formation, reaction pathway analysis will be carried out at the molecular level in the following.

### 2.3. Analysis of the Mechanisms of CO, CO_2_, and NO_X_ Formation in the Combustion of Blended Fuels as Affected by Temperature

**Figure 9:**
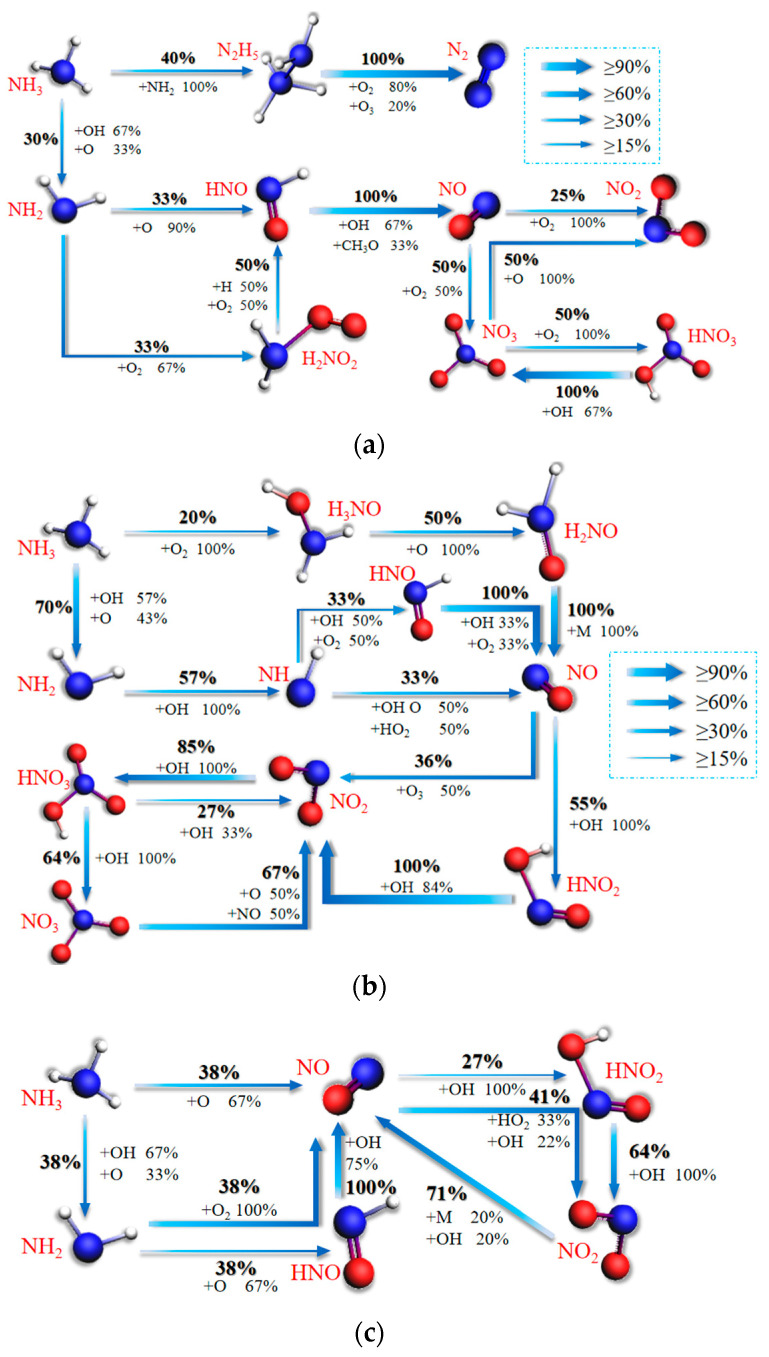
Mechanisms of NO_X_ formation from blended combustion at different temperatures. (**a**) 1000 K; (**b**) 2000 K; (**c**) 3000 K.

This section will further discuss temperature-influenced ternary blended fuel combustion in the mechanism of CO, CO_2_, and NO_X_ formation. In this section, the N and C migration paths during ternary blended fuel combustion simulated by ReaxFF MD at different temperatures are generated and discussed for Case 1, Case 2, and Case 5 as examples. Figure 9a–c represents the network diagrams for NO_X_ formation reaction paths during the combustion of ternary fuel at temperatures of 1000 K, 2000 K, and 3000 K. The percentages in the network diagrams indicate the reactant conversion rates. In order to highlight the main paths of the reaction network, reaction paths with a conversion rate of less than 15% are ignored in all network diagrams in this study.

Comparative analysis of the graphs in Figure 9 reveals that the complexity of the paths appears to be greater and then lesser as the temperature increases; the paths are most complex at 2000 K. The complexity of the paths is greater when the temperature is too low. This may be because the reaction is incomplete and molecular activity is low at 1000 K. Therefore, many molecules do not have the opportunity to collide with each other, so there are fewer intermediate products and the path is simpler. At 3000 K, because of the high temperature, the reactants are very active, especially the O molecules, so that many reactions can occur in a very short period of time. The oxidation of NH_3_ by OH during the conversion of NH_3_ molecules to NH_2_ decreases from 66.7% to less than 10%, but O increases rapidly from 33% to 67%. The intermediates required for the conversion of NH_3_ molecules to NO are gradually reduced from three to direct oxidation without intermediates. Thus, higher temperatures significantly contribute to the NH_3_ combustion reaction rate. By comparing Figure 9a–c, it is found that, as the temperature is further increased to 3000 K, the high temperature leads to the disappearance of the pathway for NH_3_ to generate NiHi, which cannot generate N_2_ but directly generates NO, and the significant increase in H and OH concentrations also contributes to the generation of NO from NH_3_ to a certain extent. This analysis validates the conclusion in Section 3.1 of this paper about heating. Although accelerating the formation of NO_X_ is not conducive to the formation of NH_3_, the conclusion is that high temperature inhibits NO_X_ formation when the temperature is higher than 2000 K. The results of this analysis are summarized in Figure 2c.

Analyzing the redox process for NO_X_, it was found that NO_X_ is all formed by NO conversion. At low temperatures (1000 K), 50% of NO is oxidized directly to NO_3_, while 25% of NO is oxidized to NO_2_. A total of 50% of NO_3_ can be reduced to NO_2_, but NO_2_ and NO_3_ cannot be reduced directly to NO. Therefore, at low temperatures, NO_X_ exists mainly as NO_3_. At 2000 K, the direct oxidation path from NO to NO_3_ disappears, and it needs to pass through NO_2_ to form NO_3_. Overall, 91% of NO is oxidized directly or indirectly to NO_2_, and the reduction of NO_3_ to NO_2_ is as high as 67%. Therefore, at 2000 K, NO_2_ is the main form of NO_X_. At high temperatures (3000 K), the pathway to generate NO_3_ disappears, and 68% of NO is oxidized directly or indirectly to NO_2_. The reduction rate for NO_2_ is as high as 71%. Therefore, NO_X_ mainly exists in the form of NO at high temperatures.

**Figure 10:**
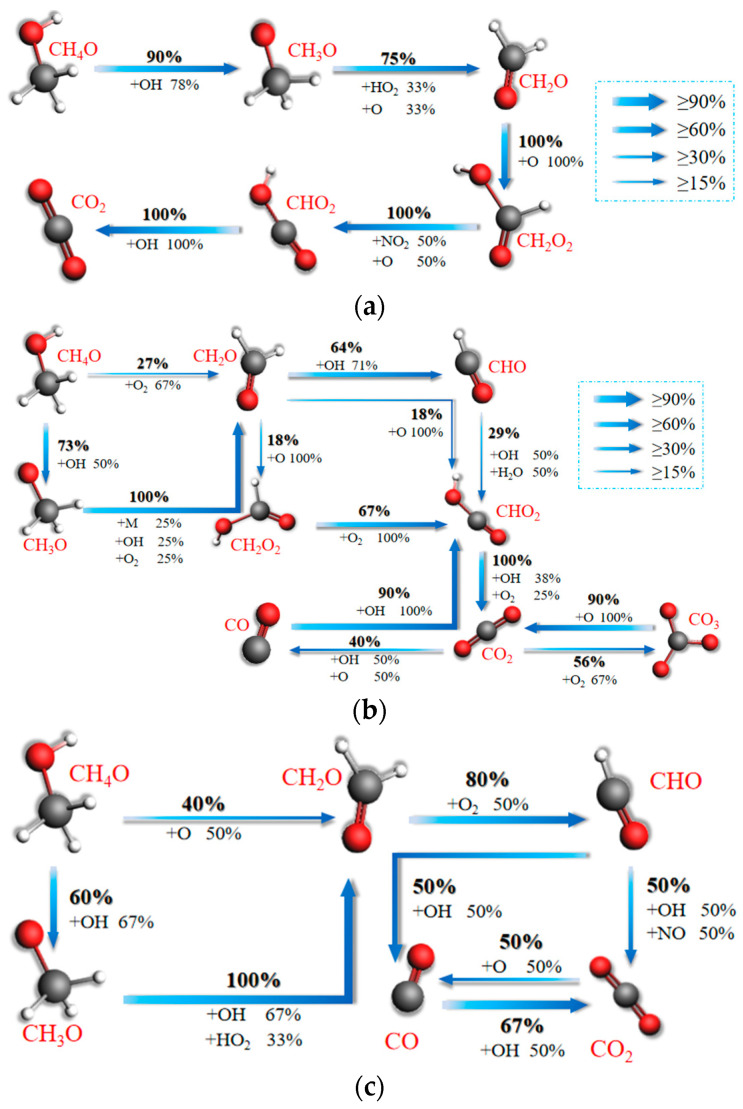
Mechanisms of CO and CO_2_ formation in the blended combustion of ternary carbon-neutral fuels at different temperatures. (**a**) 1000 K; (**b**) 2000 K; (**c**) 3000 K.

Figure 10a–c represents the network diagrams of CO_2_ formation reaction paths during combustion of ternary fuels at temperatures of 1000 K, 2000 K, and 3000 K. From Figure 10a, it can be found that 90% of CH_4_O molecules first collide with OH from O_2_ decomposition to form CH_3_O. A total of 75% of CH_3_O collides with HO_2_ and O to form CH_2_O, which is oxidized by O to form CH_2_O_2_. CH_2_O_2_ is oxidized by NO_2_ and O to form CHO_2_, which collides with OH to form CO_2_. The reaction paths are chain-shaped in this case. The path is chain-shaped and has a simple structure.

Figure 10b represents the main reaction paths of CO and CO_2_ formation by combustion of ternary hybrid fuel at a temperature of 2000 K. It can be seen that 73% of CH_4_O molecules will collide to form CH_3_O by the H_2_ extraction reaction. CH_3_O continues to collide with OH and O_2_ to form CH_2_O by a dehydrogenation reaction, while 27% of CH_4_O is oxidized by O_2_ to form CH_2_O. Unlike at 1000 K, the path from CH_2_O to CHO_2_ has expanded by two pathways: 64% of CH_2_O will collide with OH to form CHO first, and then collide with groups such as OH or H_2_O to form CHO_2_, while 18% of CH_2_O collides directly with O to form CHO_2_. The proportion of CH_2_O_2_ formation through collision with O to form CHO_2_ and then CHO_2_ (as at 1000 K) has decreased from 100% to 18%. CHO_2_ collides with groups such as OH and O_2_ to form CO_2_. CO_2_ is formed in the presence of groups such as OH, O_2_, O, and so forth. CO and CO_3_ ultimately flow to CO_2_. CO_2_ remains relatively stable in the form of an end product.

Figure 10c shows the main reaction paths for CO_2_ formation from the combustion of ternary hybrid fuels at a temperature of 3000 K. It is found that 60% of CH_4_O reacts with OH to form CH_3_O at the beginning of the combustion process at a temperature of 3000 K, and 40% of CH_4_O is directly formed into CH_2_O under the action of O. The proportions of CH_4_O reacting with OH to form CH_3_O at temperatures of 1000 K and 2000 K are 90% and 73%, respectively. The present reaction is only at 60%, and the proportions of CH_4_O directly oxidized into CH_2_O are 0 and 27% respectively, growing to 40% in the present case. Therefore, in this study, it was found that the oxidation of CH_4_O molecules during the combustion of ternary blended fuels is more pronounced as the temperature increases. This is mainly due to the fact that as the temperature increases the ternary fuel combustion reaction contains more free OH and O. The temperature also means the reactant movement is more violent, allowing more molecules to collide and participate in the pyrolysis reaction. A temperature of 3000 K generated 80% of CH_2_O by direct O oxidation to CHO; CHO and OH collision generated under half of the formation of CO and half of the formation of CO_2_.

Through further comparative analysis, it was found that with an increase in temperature, as for the N migration path, the complexity of the C migration path appeared to become larger and then smaller. The path is most complex at 2000 K. The consumption of CH_4_O molecules decreases the percentage of flow to CH_3_O from 90% to 60%, a decrease of 30%. The proportion of flow to CH_2_O increases from 0 to 40%, an increase of 40%. Thus, higher temperatures significantly contribute to the NH_3_ combustion reaction rate. By comparing Figure 10a–c, it is found that the percentage of CO formation is very small at low temperatures. At 2000 K, a CO formation pathway emerges from the reduction of 40% CO_2_ and the combination of 90% of this with OH to form CHO_2_. At a high temperature, unlike at low and medium temperatures, CO_2_ is not the only source of CO, which is not only derived from 50% CO_2_, but also from 50% CHO. In addition, the consumption of CO also decreases from 90% to 67%. Therefore, with further increases in temperature, the rate of CO and CO_2_ formation rises, and the CO peak increases. Reaction path analysis explains the phenomenon in Section 3.1 of this paper that heating accelerates the rate of CO and CO_2_ formation and the increase in peak CO with increasing temperature.

### 2.4. Mechanism and Reaction Path Analysis of CO, CO_2_, and NO_X_ Formation in Blended Fuel Combustion as Affected by Blending Ratio

**Figure 11:**
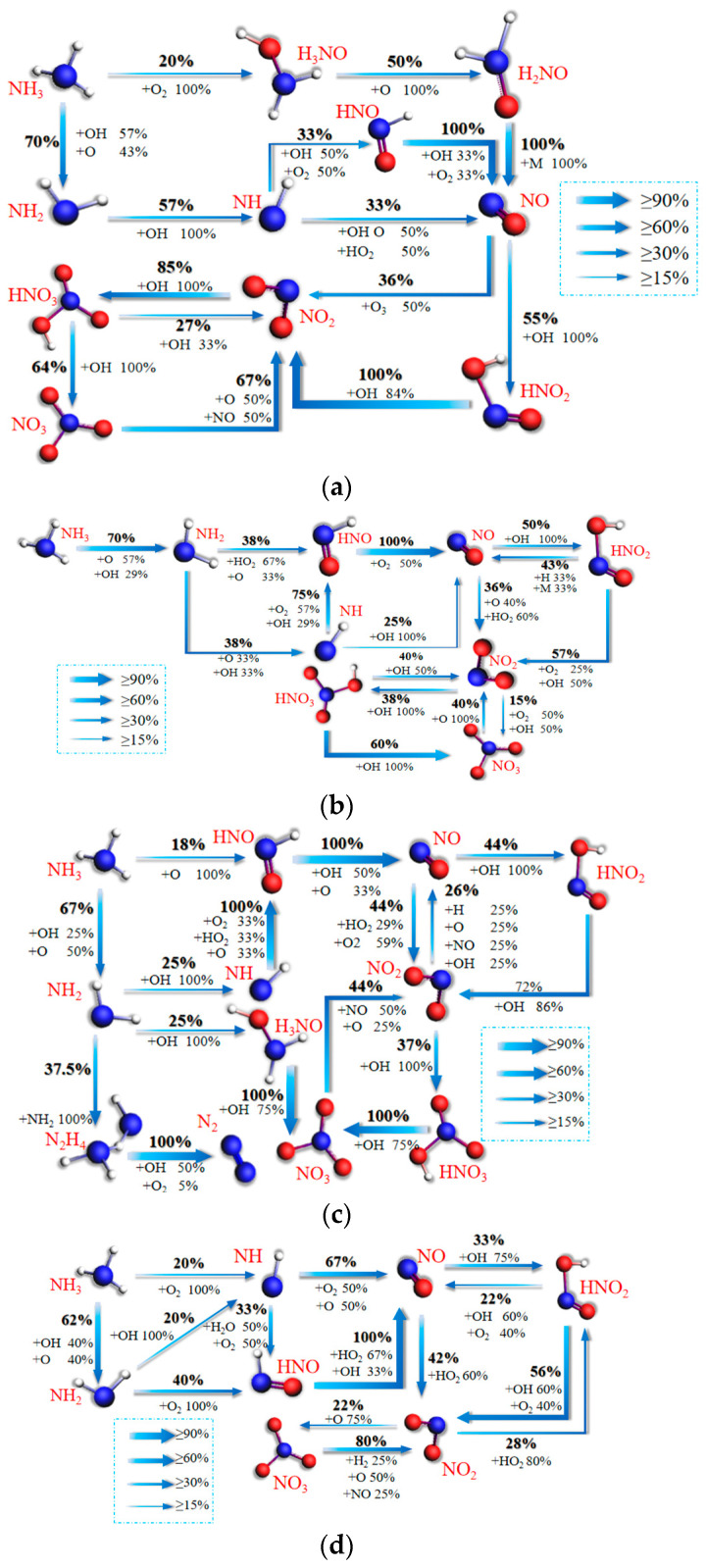
Mechanisms of NO_X_ formation from the blended combustion of ternary carbon-neutral fuels with different blending ratios. (**a**) 2:2:2; (**b**) 1:2:3; (**c**) 1:3:2; (**d**) 2:3:1.

This section will further discuss the influence of blending ratio in the combustion of ternary blended fuel on the mechanisms of CO, CO_2_, and NO_X_ formation. In this paper, the N and C migration paths of ternary blended fuel combustion are simulated by ReaxFF MD with different blending ratios generated and discussed for Case 1, Case 6, Case 7, and Case 9 as examples. Figure 11a–d represents the NO_X_ formation reaction path network diagrams during the combustion of ternary fuels with H_2_/NH_3_/CH_4_O blending ratios of 2:2:2, 1:2:3, 1:3:2, and 2:3:1.

From Figure 11a, it can be found that NH and H_3_NO generate NO by oxidizing to produce HNO and H_2_NO. A total of 55% of the NO collides with OH to produce HNO_2_, whose continued collision with OH leads to the formation of NO_2_. Meanwhile, 36% of NO is oxidized directly to NO_2_. A total of 85% of the NO_2_ collides with OH to produce HNO_3_, and 64% of the HNO_3_ is dehydrogenated to produce NO_3_ before it is reduced to NO_2_ by NO, while 27% of the HNO_3_ is directly reduced back to NO_2_ by collision with OH. A total of 25% of the NO_2_ remains relatively stable in the form of an end product.

Figure 11b shows the main reaction pathways for the formation of NO_X_ from blended combustion with a ratio of 1:2:3. HNO is oxidized to NO by O_2_, while 25% of NH is directly oxidized to NO by collision with OH. Subsequently, 14% of the NO remains relatively stable as an end product; 50% of the NO collides with OH to form HNO_2_, which is then reduced to NO_2_ by the continued collision with OH and O_2_; 36% of the NO is directly oxidized to NO_2_; and 38% of the NO is dehydrogenated with OH to form HNO_3_. A total of 60% of the HNO_3_ is dehydrogenated to NO_3_ and then reduced to NO_2_ by O. A total of 40% of HNO_3_ is dehydrogenated to NO_3_ before being reduced to NO_2_ by O.

Figure 11c shows the main reaction paths of NO_X_ formation for blended combustion with a ratio of 1:3:2. A total of 37.5% of the NH_2_ collides and combines with NH_2_ groups to form N_2_H_4_. It continues to collide with groups such as OH, O_2_, etc., to eventually form N_2_. Meanwhile, 25% of the NH_2_ collides with OH in a dehydrogenation reaction to form NH. NH collides with oxidizing groups such as O_2_, HO_2_, and O to form HNO. HNO collides with OH to form NO. A total of 12% of the NO remains relatively stable as an end product; 75.68% of the NO is oxidized to NO_2_; 37% of the NO_2_ combines with OH to form HNO_3_. HNO_3_ continues to collide with OH to form NO_3_. A total of 44% of the NO_3_ is reduced to NO_2_ by groups such as O and OH, but HNO_3_ is not the only source of NO_3_. This is because 25% of NH_2_ forms NO_3_ through H_3_NO as well.

Figure 11d represents the main reaction pathways for NO_X_ formation in blended combustion with a ratio of 2:3:1. HNO is oxidized to NO by groups such as HO_2_ and OH. A total of 20% of the NH_2_ collides with OH to form NH; 67% of the NH is oxidized directly to NO by groups such as O and O_2_; 25% of the NH is oxidized directly to NO by collisions with OH. In total, 33% of the NO and of the OH produce NO_2_ indirectly via HNO_2_, while 42% of the NO is oxidized directly to NO_2_. Overall, 22% of the NO_2_ collides with OH to produce NO_3_; 80% of the NO_3_ is reduced directly back to NO_2_ by collisions with O and NO.

Comparison of Figure 11a,b reveals that the main path of NO_X_ formation does not change much when the amount of NH_3_ fuel is the same. The reaction path is simpler at 1:2:3 compared to the 2:2:2 blending ratio. The conversion ratio of NH_3_ to NO also increases from 36.3% to 53.2%. This indicates that CH_4_O makes the NH_3_ reaction path simpler and NO_X_ formation easier. This is consistent with the conclusion in Section 3.2 that CH_4_O promotes NH_3_ combustion. Comparison of Figure 11a,c reveals that when the amount of CH_4_O fuel is certain, the proportion of NH_3_ is greater in the case of the ratio 1:3:2, which increases the reaction of NiHi to produce N_2_ and NO_3_. The conversion of NH_3_ to NO_X_ increases from 36.3% to 51.5%. The conclusion that NO_X_ is mainly determined by NH_3_ is confirmed. This conclusion is similarly confirmed in the comparative analysis of Figure 11a,d. The 2:3:1 pathway is simpler when the number of H_2_ fuels is the same. The conversion of NH_3_ to NO does not require a stepwise dehydrogenation reaction, and the conversion efficiency is increased to 50.6%.

**Figure 12:**
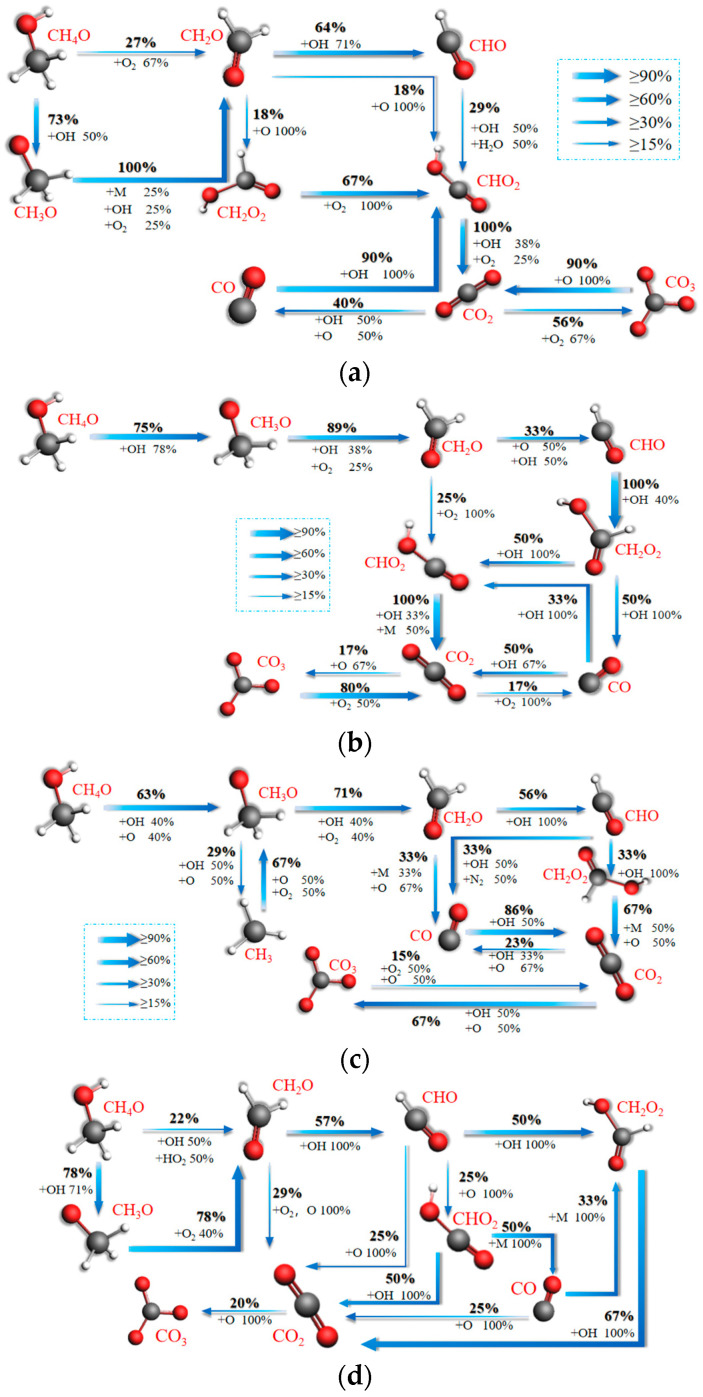
Mechanisms of CO and CO_2_ formation in the blended combustion of ternary carbon-neutral fuels with different blending ratios. (**a**) 2:2:2; (**b**) 1:2:3; (**c**) 1:3:2; (**d**) 2:3:1.

Figure 12a–d shows the network diagrams of CO_2_ formation reaction paths during the combustion of ternary fuels with H_2_/NH_3_/CH_4_O blending ratios of 2:2:2, 1:2:3, 1:3:2, and 2:3:1. Figure 12a shows the main reaction paths for the formation of CO and CO_2_ in blended combustion with a ratio of 2:2:2. CHO_2_ collides with groups such as OH and O_2_ to form CO_2_. The CO and CO_3_ formed by CO_2_ in the presence of groups such as OH, O_2_, and O ultimately flow back to CO_2_. The CO_2_ remains relatively stable in the form of an end product.

Figure 12b represents the main reaction path for the formation of CO and CO_2_ from blended combustion at a ratio of 1:2:3. It can be seen that CHO_2_ collides with OH and other groups to generate CO_2_. CO_2_ is formed under the action of OH, O_2_, O and other groups of CO and CO_3_. Compared with the different times at 2:2:2, CH_2_O_2_ can collide directly with OH to generate CO at this ratio, and the reaction path is more complex.

Figure 12c shows the main reaction paths for CO and CO_2_ formation in blended combustion when the ratio is 1:3:2. Overall, 33% of CH_2_O collides with free radicals, such as O, to form CO; 56% of CH_2_O collides with OH to form CHO; and the two portions of 33% of CHO collide with OH to form CH_2_O_2_ and CO, respectively.

Figure 12d shows the main reaction paths for CO and CO_2_ formation from the combustion of ternary blends at a ratio of 2:3:1. It is found that 78% of CH_4_O starts to react with OH and O to form CH_3_O at the ratio of 2:3:1. Overall, 78% of CH_3_O forms CH_2_O in the presence of O_2_; 57% of CH_2_O collides with free radicals such as OH to form CHO; 29% of CH_2_O collides with O_2_ to form CO_2_; and CHO generates CO and CO_2_ directly or indirectly.

Comparison of Figure 12a,b shows that when the amount of NH_3_ fuel is fixed, more CH_4_O is converted to CH_2_O with a ratio of 1:2:3 compared to 2:2:2. This may be due to the fact that H_2_ promotes the oxidation of CH_4_O. There were more reaction pathways for CO and CO_2_ formation compared to the 2:2:2 ratio of 1:2:3. This may be caused by the high number of OH radicals in the reaction.

Comparison of Figure 12a,c reveals that the two paths do not differ much when the amount of CH_4_O fuel is the same. This indicates that the reactions of CO and CO_2_ during the combustion of ternary fuels are mainly influenced by CH_4_O.

Comparison of Figure 12a,d reveals that when the number of H_2_ fuels is the same, the path with a ratio of 2:3:1 is more complex. However, it does not mean that the oxidation of carbon-containing fuels in ternary fuels is more intense. Analyzing the percentage of oxidizing groups in the pathway reveals that, on the contrary, it is the oxidation of carbon-containing fuels at 2:3:1 that does not have enough O and OH groups. This time more intermediate OH groups are needed. From the analysis of OH groups mainly influenced by CH_4_O it can be concluded that CO and CO_2_ are mainly determined by CH_4_O.

## 3. Materials and Methods

### 3.1. Reactive Force-Field Molecular Dynamics (ReaxFF MD)

ReaxFF MD combines molecular dynamics simulation with the calculation of reactive force fields. Its reactive force-field potential function is derived from experimental data and density functional theory, so the accuracy is close to quantum computation and does not require the predetermination of chemical reaction paths in the system [32]. ReaxFF is parameterized against QM-based training sets and is dependent on the bond order, while the bond order is a function of interatomic distance and updates at every iteration. Therefore, ReaxFF can describe bond formation and dissociation and provide highly accurate simulation results. ReaxFF MD has been widely used in the study of pyrolysis [33], combustion [34], explosions [35], oxidation [36], catalysis [37], and other systems involving physical chemistry. It provides a promising means of exploring the chemical behavior of complex molecular systems. Bond-order-dependent characterization is achieved by detailed parameterization of the atomic, bonding, angular, and torsional properties of each particle, and the interactions within the system [38]. The total energy of the system can be calculated by summing all partial energy terms as described in R1:

E_system_ = E_bond_ + E_over_ + E_under_ + E_val_ + E_pen_ + E_tors_ + E_conj_ + E_vdWaals_ + E_coulomb_ (1)

where E_bond_, E_over_, E_under_, E_val_, E_pen_, E_tors_, and E_conj_ correspond to bond energy, over-coordination energy, under-coordination energy, bond angle energy, compensation energy, torsion energy, and four-body conjugation energy. The non-bonding terms mainly consist of van der Waals force energy (E_vdWaals_) and Coulomb force energy (E_coulomb_). When calculating non-bonding interactions, the charged atoms cross the truncation radius of the non-bonding interactions, thus leading to a jump in energy. Therefore, ReaxFF is additionally corrected by introducing a seventh-order polynomial Taper function, which ensures that, at the truncation radius, the non-bonding interaction’s first-, second-, and third-order derivatives of the energy term are all zero [39]. ReaxFF also takes better account of charge polarization by employing the electronegativity equalization method [40] and updates the atomic charges at each time step [41]. The detailed meaning of the ReaxFF parameters, the setup of the molecular structure, and the applicability of the reaction force field have been described in detail in a previous study [42].

### 3.2. Case Set-Ups

Table 1 lists all H_2_/NH_3_/CH_4_O blend combustion ReaxFF MD simulation cases in the high-pressure environment of this paper. The system density (ρ), equivalence ratio (φ), and simulation time are 0.05 g/cm^3^, 0.5, and 1.25 ns, where more air (φ = 0.5) is used to ensure complete fuel combustion. Cases 1 to 5 represent the combustion of H_2_/NH_3_/CH_4_O blended fuel at 2000 K, 1000 K, 1500 K, 2500 K, and 3000 K. Cases 6 to 11 represent combustion at the same temperature with H_2_/NH_3_/CH_4_O ratios of 1:2:3, 1:3:2, 2:1:3, 2:3:1, 3:1:2, and 3:2:1. Three replicates with different initial configurations were simulated for individual cases. All the results reported in this work are ensemble-averaged from them. Through further comparative analysis, the mechanisms of CO, CO_2_, and NO_X_ formation at different temperatures and fuel ratios are analyzed at the molecular level.

### 3.3. Computational Details and Post-Processing

**Figure 13:**
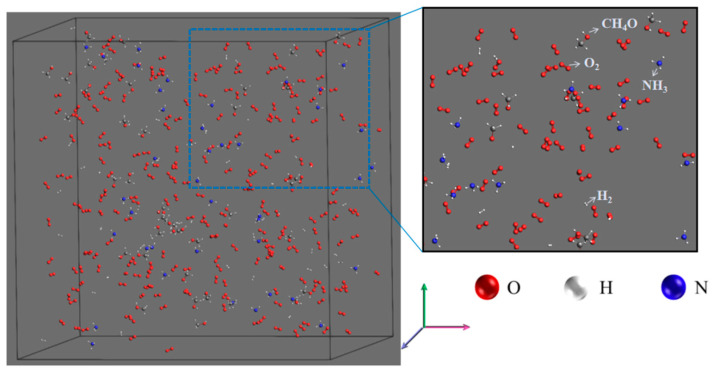
Optimization system for Case 1.

All the cases listed in Table 1 were analyzed in the ReaxFF module of AMS [43,44,45]. In this study, the HE2.ff force field [46] and the regular system with constant atomic number, volume, and temperature (NVT) were used. To ensure the overall stability of hydrocarbon fuel combustion, the energy and configuration of all simulated cases were first optimized using the “Geometry Optimization” and “Energy Optimization” plugins. Figure 13 shows the optimized systematic for Case 1, which shows that fuel and oxidant are uniformly blended, similar to a premixed flame, and similar to the cyclone burner we previously employed [47]. A Berendsen thermostat was used to control the temperature with a time step of 0.25 fs. Periodic boundary conditions were applied in all three xyz directions and the soot intermediate components and product distributions were analyzed from trajectories using a 0.3 Å bond level cutoff. All simulations were done on a server with an Intel(R) Xeon(R) Platinum 8352Y CPU @ 2.20 GHz, 64-core CPU, and 256 GB of RAM, and each set of conditions simulated for 1 ns required approximately 30 h of CPU time.

### 3.4. Validation of the ReaxFF MD Method

The reliability and validity of the ReaxFF MD method have been widely tested and verified in previous studies [38,39,48,49,50,51]. Among them, Wang et al. [39] constructed the reaction pathways in high-pressure combustion by tracking the trajectories of reacting atoms through ReaxFF MD to understand NO_X_ formation mechanisms in NH_3_/CH_4_ combustion at different temperatures and pressures. The results showed that a high temperature accelerated the rate of ammonia consumption, which was consistent with the experimental results. High pressure complicated the reaction pathway in NH_3_/CH_4_ combustion with the emergence of new intermediates and primitive reactions. In addition, they pointed out that ReaxFF MD is a valuable tool to reveal the underlying reaction mechanisms in combustion and pollutant formation. Liu et al. [51] investigated the chemical reactivity effects of NO on the oxidation of CH_4_ using ReaxFF MD simulations and found that increasing the blending ratio of NO accelerated the rate of CH_4_ consumption. This is mainly due to the fact that, on the one hand, conversion of NO to NO_2_ generates OH radicals, which accelerates CH_4_ consumption, while, on the other hand, NO can also inhibit CH_4_ consumption by combining with reactive radicals. Wang et al. [48] applied ReaxFF MD and Py-GC/MS to investigate the characteristics of soot particulate formation in the process of the hydrogen-doped combustion of methane and ethylene. Both experimental and numerical results reflected that PAHs and ethylene were not the most important pollutants in the combustion process of CH_4_. The experimental and numerical results reflect the evolution of PAHs and initial soot particles, as well as the different chemical effects of hydrogen doping on PAHs and soot formation.

## 4. Conclusions

In this paper, the effects of different reactant temperatures and blending ratios on combustion reaction rates and the formation characteristics of CO, CO_2_, and NO_X_ in the combustion of H_2_/NH_3_/CH_4_O ternary carbon-neutral blended fuels were investigated for the first time using ReaxFF MD. The mechanisms of CO, CO_2_, and NO_X_ formation in ternary blended fuels at different temperatures and blending ratios were investigated. The conclusions of this paper are summarized as follows:

(1) Heating accelerates the rate of H_2_, NH_3_, CH_4_O, and O_2_ consumption during ternary fuel combustion. However, the effect of heating on products such as N_2_ and H_2_O is not linear. The lowest amount of N_2_ was produced and the most amount of NO_X_ was generated at 2000 K. The reaction rate and formation of CO and CO_2_ increased with temperature. The CO peak shifted forward with increasing temperature. Free radical analysis revealed that CO, CO_2_, and NO_X_ may be closely related to large amounts of OH. The predominant form present in NO_X_ changed with temperature. However, CO_2_ has been the main form present in carbonaceous pollutants.

(2) Pollutant formation during the combustion of H_2_/NH_3_/CH_4_O ternary carbon-neutral blends was influenced by the coupling of H_2_, NH_3_, and CH_4_O. NH_3_ suppressed the CO formation rate when the percentage of CH_4_O was greater than 30%. However, the amount of CO and CO_2_ formation was mainly determined by CH_4_O, which increased the NH_3_ combustion rate, causing NH_3_ combustion to form more NO_X_. In the ternary blended combustion process, NH_3_ inhibits H_2_ combustion, but CH_4_O promotes H_2_ combustion, in which NH_3_ plays a major role.

(3) Analysis of the formation mechanisms of pollutants from the combustion of ternary carbon-neutral blended fuels at different temperatures reveals that high temperatures lead to more active oxidizing groups such as O in the reaction, which inhibits N_2_ formation. The pathway of NO_X_ is more complicated at 2000 K. NO_X_ is formed by the conversion of NO. At low temperatures, half of the NO is oxidized directly to NO_3_, but NO_2_ and NO_3_ cannot be reduced to NO directly. At 2000 K, NO needs to pass through NO_2_ to form NO_3_. A large amount of NO is oxidized to NO_2_, and the reduction of NO_3_ to NO_2_ is higher. At high temperatures, the pathway to generate NO_3_ disappears, and NO_2_ has a higher reduction rate. Therefore, the main form of NO_X_ exists differently in different temperature states. Higher temperatures lead to the emergence of more CO formation paths, making CO and CO_2_ be produced more quickly.

(4) By analyzing the formation mechanisms of pollutants in the combustion of ternary carbon-neutral blends with different blending ratios, it was found that CH_4_O promotes the combustion of NH_3_. This makes the reaction path simpler and easier for the generation of NO_X_. CH_4_O not only provides more carbon atoms involved in collisions for CO and CO_2_ formation, but also leads to more OH and H formation. Therefore, the amount of CO and CO_2_ formation is mainly determined by CH_4_O.

## Figures and Tables

**Table 1:** ReaxFF MD cases of H_2_/NH_3_/CH_4_O blended combustion.

Case	H_2_	NH_3_	CH_4_O	O_2_	ρ, g/cm^3^	T, K	φ
1	40	40	40	220	0.05	2000	0.5
2	40	40	40	220	0.05	1000	0.5
3	40	40	40	220	0.05	1500	0.5
4	40	40	40	220	0.05	2500	0.5
5	40	40	40	220	0.05	3000	0.5
6	20	40	60	260	0.05	2000	0.5
7	20	60	40	230	0.05	2000	0.5
8	40	20	60	250	0.05	2000	0.5
9	40	60	20	190	0.05	2000	0.5
10	60	20	40	210	0.05	2000	0.5
11	60	40	20	280	0.05	2000	0.5

## Data Availability

Data are contained within the article.

## Abbreviations

AMS	Amsterdam Modeling Suite	N_2_	Nitrogen
CH_4_O	Methanol	NH_3_	Ammonia
CH_3_O	Methoxy	NH_2_	Ammonia radical
CH_2_O	Formaldehyde	NO	Nitric oxide
CNG	Compressed natural gas	NO_2_	Nitrogen dioxide
CO	Carbon monoxide	NO_3_	Nitrogen trioxide
CO_2_	Carbon dioxide	NOx	Nitrogen oxide
DGE	Diethylene glycol ether	NVT	Constant number of atoms, constant volume, and controlled temperature
H_2_	Hydrogen	ReaxFF MD	Reactive molecular dynamics simulation
HC	Total hydrocarbons	PAH	Polycyclic aromatic hydrocarbons
HNO	Nitric acid	PM	Particulate matter
HO_2_	Hydrogen peroxide radical	φ	Equivalent ratio
LNG	Liquefied natural gas	ρ	System density
